# HIV-1 Genetic Characteristics and Transmitted Drug Resistance among Men Who Have Sex with Men in Kunming, China

**DOI:** 10.1371/journal.pone.0087033

**Published:** 2014-01-29

**Authors:** Min Chen, Yanling Ma, Yingzhen Su, Li Yang, Renzhong Zhang, Chaojun Yang, Huichao Chen, Wenyun Yan, Yuhua Shi, Lijuan Dong, Ling Chen, Manhong Jia, Lin Lu

**Affiliations:** 1 Center for AIDS/STD Control and Prevention, Yunnan Center for Disease Control and Prevention, Kunming, Yunnan, China; 2 College of Public Health, Kunming Medical University, Kunming, Yunnan, China; Johns Hopkins School of Public Health, United States of America

## Abstract

**Background:**

Yunnan has been severely affected by HIV/AIDS in China. Recently, the reported prevalence of HIV-1 among men who have sex with men (MSM) in Yunnan was high in China. To monitor dynamic HIV-1 epidemic among Yunnan MSM, HIV-1 genetic characteristics and transmitted drug resistance (TDR) were investigated.

**Methods:**

Blood samples from 131 newly HIV-1 diagnosed MSM were continuously collected at fixed sites from January 2010 to December 2012 in Kunming City, Yunnan Province. Partial *gag*, *pol* and *env* genes were sequenced. Phylogenetic, evolutionary and genotypic drug resistance analyses were performed.

**Results:**

Multiple genotypes were identified among MSM in Kunming, including CRF01_AE (64.9%), CRF07_BC (25.2%), unique recombinant forms (URFs, 5.3%), subtype B (3.1%) and CRF08_BC (1.5%). CRF01_AE and CRF07_BC were the predominant strains. The mean of genetic distance within CRF01_AE were larger than that within CRF07_BC. The estimated introducing time of CRF01_AE in Yunnan MSM (1996.9) is earlier than that of CRF07_BC (2002.8). In this study, subtype B was first identified in Yunnan MSM. CRF08_BC seems to be the distinctive strain in Yunnan MSM, which was seldom found among MSM outside Yunnan. The proportion of URFs increased, which further contributed to genetic diversity among MSM. Strikingly, genetic relatedness was found among these strains with MSM isolates from multiple provinces, which suggested that a nationwide transmission network may exist. TDR-associated mutations were identified in 4.6% individuals. The multivariate analysis revealed that non-native MSM and divorced/widowed MSM were independently associated with a higher TDR rate.

**Conclusion:**

This work revealed diverse HIV-1 genetics, national transmission networks and a baseline level of TDR in MSM. These findings enhance our understanding of the distribution and evolution of HIV-1 in MSM, and are valuable for developing HIV prevention strategies for MSM.

## Introduction

The term MSM refers to behavior rather than identity or sexual orientation, and covers a large variety of settings and contexts in which male-to-male sex takes place. MSM include men who share a non-heterosexual identity and men who view themselves as heterosexual but who engage in sex with other males for various reasons. Since Acquired Immunodeficiency Syndrome (AIDS) was first described in homosexual men in 1981 [1], MSM have been mostly affected by human immunodeficiency virus (HIV) worldwide [2], [3]. A review of available data from 2007 to 2011 showed that HIV prevalence in MSM ranged from 3.0% in the Middle East and North Africa region to 25.4% in the Caribbean, and HIV infection levels in MSM were substantially higher than those in non-MSM individuals [4].

In recent years, a fast-spreading HIV epidemic among MSM constitutes a new challenge in China. Between 2006–2011, the annual rate of newly reported HIV cases attributed to homosexually transmitted infection in China increased from 2.5% to 13.7% [5]. HIV prevalence from MSM sentinel surveillance data also showed a rising trend from 0.9% in 2003 to 6.3% in 2011 [6]. A meta-analysis found national HIV prevalence among Chinese MSM has increased from 1.4% in 2001 to 5.3% in 2009 [7]. In 2008, a cross-sectional study from 18,000 MSM in 61 cities of China found an average HIV prevalence of 4.9% with incidence ranging between 2.6 to 5.4 per 100 person-years [8]. These studies suggested that MSM are an important target population for HIV prevention in China.

Yunnan is located in southwest China and situated along the drug trafficking routes channeling heroin into China. Since the first HIV epidemic in China was identified among intravenous drug users (IDUs) in Yunnan in 1989, Yunnan has been one of the areas hardest hit by HIV in China [9]. By the end of 2011, the cumulative number of reported HIV/AIDS in Yunnan was 95296, accounting for 21.0% of the total national figure [5]. Initially, HIV epidemic in Yunnan was driven by IDUs. After 2006, the main transmission route changed from intravenous injection to sexual contact. Besides heterosexually transmitted infection, the HIV epidemic in MSM is of particular concern. During 2007–2008, the prevalence of HIV among MSM in Kunming (the capital city of Yunnan) reached 10.83% [10]. A recent meta-analysis indicated that MSM in Southwest China had the highest HIV prevalence, of 11.4% in comparison with other regions, which ranged between 3.5% and 4.8% [7]. These suggested that Yunnan bears higher HIV burden in the MSM population.

Yunnan was considered as an entrance of different HIV-1 genotypes into China. In the late 1980s and early 1990s, subtype B and C were introduced into Yunnan from Myanmar and India by IDUs [9]. In 1994, CRF01_AE was identified among commercial sex workers returning from Thailand to Yunnan [11]. Thereafter, CRF07_BC and CRF08_BC were initially established in Yunnan [12], [13] and spread through two different overland heroin trafficking routes: CRF07_BC northwestward to Xinjiang [14], [15], and CRF08_BC eastward to Guangxi [16], [17]. Nowadays, HIV-1 genetic diversity is a prominent feature of Yunnan’s HIV-1 epidemic [18]–[21]. CRF08_BC was the most common genotype in Yunnan, followed by URFs, CRF01_AE, CRF07_BC, subtype B and subtype C [21].

To date, there is no comprehensive HIV molecular epidemiological study in Yunnan which provides insights into transmission and acquisition risks, transmission dynamics, and challenges to HIV prevention for MSM. Currently, early antiretroviral therapy is considered as a prevention strategy in high-risk groups, because it can limit transmission of HIV by reducing viral replication. The TDR level among antiretroviral therapy (ART)-naïve MSM will affect the efficiency of this intervention. An overall 4.9% TDR rate was found among ART-naïve MSM in 19 provinces/cities in China in 2010 [22]. However, the TDR rate among ART-naïve MSM in Yunnan has not yet been thoroughly studied.

In the present study, we conducted a large-scale HIV molecular epidemiological and TDR survey among MSM in Kunming City, where both the estimated number of MSM and the reported number of HIV-positive MSM are more than half of the counterparts of the whole province. The findings would be valuable to better understand the HIV epidemic and develop intervention strategies for MSM individuals in Yunnan.

## Materials and Methods

### Study Participants and Sample Collection

A total of 131 newly confirmed HIV-1-positive MSM blood samples were continuously collected between January 2010 and December 2012 through fixed voluntary counseling and testing sites (VCT) and non-government organizations in Kunming City, Yunnan Province. HIV-1 infection status was determined by an Enzyme-Linked Immunosorbent Assay (ELISA, Biomerieux, France) and confirmed by Western blot assay (HIV BLOT 2.2, MP Diagnostics, Singapore). All HIV tests were informed and voluntary. Written consents were obtained from all participants. The study was approved by Biomedical Ethics Review Committee of Yunnan Province.

### Amplification of HIV-1 Gene Fragments

Plasma was separated from whole blood. Viral RNA was extracted from 140 µl of plasma by using the QIAamp Viral RNA Mini kit (Qiagen, Valencia, CA, United States) according to the manufacturer’s instructions, and was then subjected to nested polymerase chain reaction (PCR) to generate the fragments of the *gag* (HXB2: 781–1861), *pol* (HXB2: 2147–3462) and *env* (HXB2: 7002–7541). The *gag* fragment was amplified using One Step reverse transcription PCR (Takara, Dalian, China) with primers GAG-L (5′- TCGACGCAGGACTCGGCTTGC -3′) and GAG-E2 (5′- TCCAACAGCCCTTTTTCCTAGG -3′) in 25 µl reaction volume. Cycling conditions were as follows: 50°C for 30 min ; 94°C for 5 min, 55°C for 1 min, 72°C for 2 min;94°C for 30 s, 55°C for 45 s, 72°C for 1 min 30 s, 30 cycles;72°C for 10 min. The nested *gag* PCR was performed using 2×Taq PCR MasterMix (Tiangen, Beijing, China) with primers GUX (5′-AGGAGAGAGATGGGTGCGAGAGCGTC-3′) and GDX (5′- GGCTAGTTCCTCCTACTCCCTGACAT-3′) in 50 µl reaction volume. Cycling conditions were: 94°C for 2 min, 55°C for 1 min, 72°C for 1 min 30 s;94°C for 30 s, 55°C for 45 s, 72°C for 1 min 30 s, 30 cycles;72°C for 10 min. The *pol* fragment was amplified with primers MAW26 (5′-TTGGAAATGTGGAAAGGAAGGAC-3′) and RT21 (5′-CTGTATTTCTGCTATTAAGTCTTTTGATGGG-3′) with cycling conditions: 50°C for 30 min;94°C for 5 min;94°C for 30 s, 55°C for 30 s, 72°C for 2 min 30 s, 30 cycles;72°C for 10 min. The nested *pol* PCR was performed with primers PRO-1 (5′-CAGAGCCAACAGCCCCACCA-3′) and RT20 (5′-CTGCCAGTTCTAGCTCTGCTTC-3′) with cycling conditions as following: 94°C for 5 min;94°C for 30 s, 63°C for 30 s, 72°C 2 min 30 s, 30 cycles;72°C for 10 min. The *env* fragment was amplified with primers 44F (5′-ACAGTRCARTGYACACATGG-3′) and 35R (5′-CACTTCTCCAATTGTCCITCA-3′) with cycling conditions: 50°C for 30 min;94°C for 2 min, 50°C for 1 min, 72°C for 4 min;94°C for 30 s, 55°C for 30 s, 72°C for 2 min, 30 cycles;72°C for 10 min. The nested *env* PCR was performed with primers 33F (5′- CTGTTIAATGGCAGICTAGC -3′) and 48R (5′- RATGGGAGGRGYATACAT -3′) with cycling conditions as: 95°C for 2 min; 95°C for 15 s, 55°C for 30 s, 72°C 1 min 15 s, 5 cycles;95°C for 15 s, 60°C for 30 s, 72°C for 1 min, 25 cycles; 72°C for 10 min. The generated products were analyzed using 1% agarose gel electrophoresis. Positive samples were sent to ZIXIBIO Co. (Beijing, China) for sequencing using an ABI 3730XL automated DNA sequencer (Applied Biosystems, Carlsbad, USA) with the following primers: *gag*: GUX/GDX; *env*: 33F/48R; *pol*: PROS3 (5′-GCCAACAGCCCCACCA-3′), RTAS (5′-CTCAGATTGGTTGCAC-3′), RTB (5′-CCTAGTATAAACAATGAGACAC-3′), PROC1S (5′-GCTGGGTGTGGTATTCC-3′), and RT20S3 (5′-GTTCTAGCTCTGCTTC-3′).

### Sequence Analysis

The contig assembly of sequences was performed using DNA sequence analysis software Sequencher 5.0 (Gene Codes, Ann Arbor, MI). The ClustalW Multiple alignment and manual editing were performed using Bio-Edit 7.0 software. The reference sequences were obtained from the NIH/NIAID-funded HIV Databases (http://hiv-web.lanl.gov/content/index), covering the major HIV-1 subtypes/CRFs. Some sequences of MSM or other high-risk groups previously identified in China and Yunnan were included (Table S1). Phylogenetic tree analyses were performed using the neighbor-joining method based on Kimura two-parameter model with 1000 bootstrap replicates, using MEGA (Molecular Evolutionary Genetics Analysis, version 5.1) [23].

For subtype B, CRF01_AE and CRF07_BC, genetic distances of pair sequences within each genotype were calculated on *gag*, *pol* and *env* fragments using Kimura two-parameter model. To demonstrate possible intersubtype mosaicism, candidate sequences were analyzed using the Recombination Identification Program (RIP, version 3.0; http://hiv-web.lanl.gov), and were further analyzed with similarity plot analyses using program Simplot (version 3.5.1; S. Ray, Johns Hopkins University, Baltimore, MD) [24].

The HIV-1 genotype of each patient was assigned based on the genotypes of the *gag*, *pol* and *env* genes; if one of the three genes was unavailable, the genotype was assigned based on the other two genes. Samples with different genotypic identification assigned to the *gag*, *pol* and *env* regions were deemed URF. The designations of URFs include the genotypes identified from the *gag*, *pol* and *env* genes, and were labeled by the sequence of *gag*/*pol*/*env*. The details were shown in the Results.

### Genotypic Analysis of HIV-1 Drug Resistance

The nucleotide sequences of *pol* gene, containing the full-length protease gene and the first 299 codons of the reverse transcriptase gene, were submitted to Stanford HIV Drug Resistance Database (http://hivdb.stanford.edu). The proportion of submitted sequences containing a mutation suggestive of transmitted HIV-1 drug resistance was determined with Calibrated Population Resistance (CPR) Tool (Version 6.0). For each transmitted drug resistant strain, the levels of resistance to commonly used proteases and RT inhibitors were analyzed with Genotypic Resistance Interpretation Algorithm.

### Bayesian MCMC Evolutionary Analyses

The evolution rate and time of most recent common ancestor (tMRCA) of CRF01_AE and CRF07_BC strains circulating among MSM in Kunming were inferred on *pol* gene using Bayesian Markov chain Monte Carlo (MCMC) method. The general time reversible (GTR) model plus a gamma distribution (Γ4) among site rate heterogeneity (I) model (GTR+I+Γ4) was evaluated as the best nucleotide substitution model for all datasets by the jModeltest version 2.1.2. Bayesian MCMC analyses were performed using a Bayesian uncorrelated exponential relaxed molecular clock method in combination with four different coalescent tree priors (‘Constant Size’; ‘Exponential Growth’, ‘Logistic Growth’ and ‘Bayesian Skyline’) under the selected nucleotide substitution model in the BEAST v1.7.4 package [25]. Each MCMC analysis was run for at least 20 million generations and sampled every 2,000 generations. The resulting log-files were analyzed in Tracer v1.5 and the Bayes Factor was calculated to compare molecular clock models, using marginal likelihood as implemented in Tracer v.1.5. The Maximum Clade Credibility (MCC) tree was obtained by TreeAnnotator v1.7.4 with a burn-in of the initial 25% of generated trees, and examined by FigTree V1.3.1, which was also used to estimate the evolutionary rates and the dates to tMRCA of various nodes on the MCC tree.

### Statistical Analysis

Statistical analyses were conducted using the SPSS 17.0 statistical analysis software package (SPSS Inc. Chicago, IL). Categorical variables were compared using χ^2^ tests. Variables in the multivariate logistic analysis were selected based on variables which were marginally significant with *p*<0.20 in univariate logistic analysis. Multivariate logistic analysis was done using a forward multiple unconditional logistic regression model in order to determine adjusted odds ratios (aOR) for risk-factors related to drug resistance. All tests were two-tailed and a *p*-value <0.05 was considered statistically significant.

## Results

### Demographic Characteristics of Study Subjects

A total of 131 newly confirmed HIV-positive MSM samples were collected in Kunming City from 2010 to 2012. Of these MSM, 34.4% (45/131) were permanent residents in Kunming, 39.7% (52/131) were from other cities in Yunnan Province, and 26.0% (34/131) were from other provinces. The median age of HIV-infected MSM was 26.0 years (range: 17–70 years); and 84.7% (111/131) of participants were of Han ethnicity, and 15.3% (20/131) of participants were of minority ethnicity, including Yi, Bai, Hui, Hani, Lisu, Menggu, Shui and Zhuang. Of all subjects, 85.5% (112/131) were single, 6.9% (9/131) were married, and 7.6% (10/131) were divorced or widowed. More than half of participants (52.7%, 69/131) had received college-level or higher education degree. Multiple occupations were reported among the study participants: students accounted for 16.0% (21/131); employees in the service industry for 41.2% (54/131); civil servants, teachers and doctors for 11.5% (15/131); workers and countrymen for 14.5% (19/131); and otherwise employed for 16.8% (22/131) (Table 1).

**Table 1:** Demographic characteristics and genotypes of study subjects.

	Total	Genotypes					?2	*P*
		B	CRF01_AE	CRF07_BC	CRF08_BC	URF		
Total	131 (100.0%)	4 (3.1%)	85 (64.9%)	33 (25.2%)	2 (1.5%)	7 (5.3%)		
Collection Time							6.536	0.548
2010	34 (26.0%)	0 (0.0%)	23 (17.6%)	8 (6.1%)	0 (0.0%)	3 (2.3%)		
2011	27 (20.6%)	0 (0.0%)	21 (16.0%)	6 (4.6%)	0 (0.0%)	0 (0.0%)		
2012	70 (53.4%)	4 (3.1%)	41 (31.3%)	19 (14.5%)	2 (1.5%)	4 (3.1%)		
Permanent Residence							6.896	0.522
Kunming City	45 (34.4%)	1 (0.8%)	31 (23.7%)	10 (7.6%)	0 (0.0%)	3 (2.3%)		
Other Cities in Yunnan Province	52 (39.7%)	0 (0.0%)	34 (26.0%)	14 (10.7%)	1 (0.8%)	3 (2.3%)		
Other Provinces	34 (26.0%)	3 (2.3%)	20 (15.3%)	9 (6.9%)	1 (0.8%)	1 (0.8%)		
Age							12.509	0.299
≤24	53 (40.5%)	2 (1.5%)	34 (26.0%)	15 (11.5%)	0 (0.0%)	2 (1.5%)		
25–34	51 (38.9%)	1 (0.8%)	33 (25.2%)	14 (10.7%)	0 (0.0%)	3 (2.3%)		
35–44	17 (13.0%)	0 (0.0%)	12 (9.2%)	2 (1.5%)	1 (0.8%)	2 (1.5%)		
≥45	10 (7.6%)	1 (0.8%)	6 (4.6%)	2 (1.5%)	1 (0.8%)	0 (0.0%)		
Nationality							2.111	0.665
Han	111 (84.7%)	4 (3.1%)	69 (52.7%)	30 (22.9%)	2 (1.5%)	6 (4.6%)		
Other	20 (15.3%)	0 (0.0%)	16 (12.2%)	3 (2.3%)	0 (0.0%)	1 (0.8%)		
Marriage Status							8.969	0.268
Single	112 (85.5%)	3 (2.3%)	75 (57.3%)	27 (20.6%)	1 (0.8%)	6 (4.6%)		
Married	9 (6.9%)	1 (0.8%)	5 (3.8%)	3 (2.3%)	0 (0.0%)	0 (0.0%)		
Divorced/Widowed	10 (7.6%)	0 (0.0%)	5 (3.8%)	3 (2.3%)	1 (0.8%)	1 (0.8%)		
Education							2.384	0.676
Compulsory education and below	29 (22.1%)	1 (0.8%)	17 (13.0%)	8 (6.1%)	1 (0.8%)	2 (1.5%)		
Compulsory education above	102 (77.9%)	3 (2.3%)	68 (51.9%)	25 (19.1%)	1 (0.8%)	5 (3.8%)		
Occupation							12.426	0.639
Students	21 (16.0%)	2 (1.5%)	12 (9.2%)	7 (5.3%)	0 (0.0%)	0 (0.0%)		
Employees in the service industry	54 (41.2%)	2 (1.5%)	31 (23.7%)	15 (11.5%)	1 (0.8%)	5 (3.8%)		
Civil Servants, Teachers and Doctors	15 (11.5%)	0 (0.0%)	10 (7.6%)	4 (3.1%)	0 (0.0%)	1 (0.8%)		
Workers and Peasants	19 (14.5%)	0 (0.0%)	14 (10.7%)	4 (3.1%)	1 (0.8%)	0 (0.0%)		
Others	22 (16.8%)	0 (0.0%)	18 (13.7%)	3 (2.3%)	0 (0.0%)	1 (0.8%)		

### HIV-1 Genotype Analysis in MSM Population

**Figure 1:**
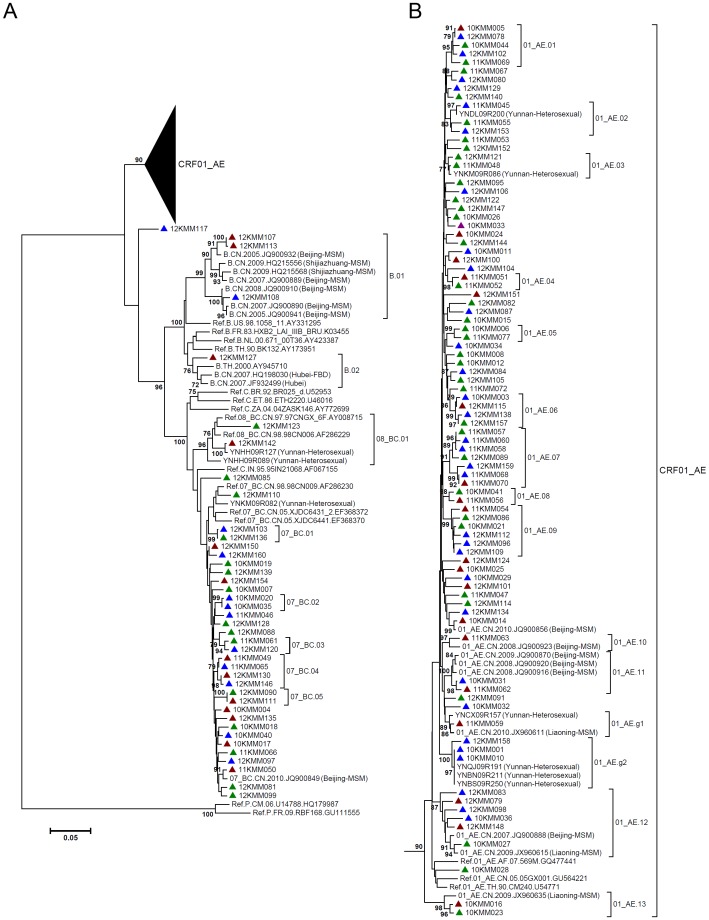
Neighbor-joining phylogenetic tree of partial *gag* gene from newly HIV-1 diagnosed MSM. A, Neighbor-joining phylogenetic tree for 126 *gag* sequences and relative reference sequences. B, CRF01_AE clade from the phylogenetic tree shown in A. Blue triangle: Kunming native MSM; green triangle: MSM form other cities in Yunnan Province; red triangle: MSM from other provinces. The sequences of MSM or other high-risk groups previously identified in China and Yunnan were included. The scale bar indicates 5% nucleotide sequence divergence. Values on the branches represent the percentage of 1000 bootstrap replicates and bootstrap values over 70% are shown in the tree.

**Figure 2:**
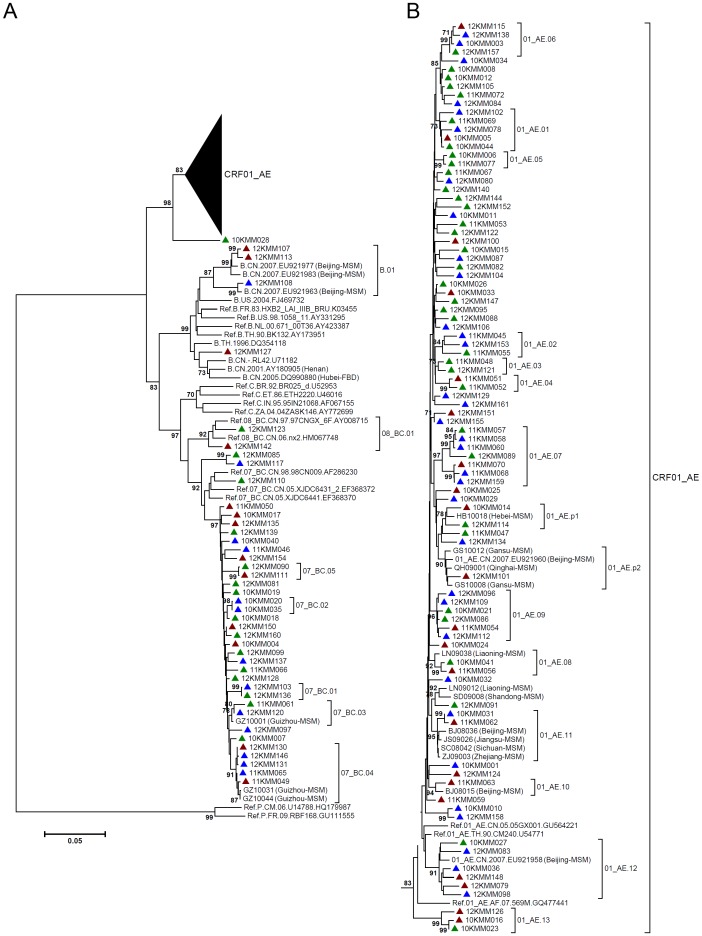
Neighbor-joining phylogenetic tree of partial *pol* genes from newly HIV-1 diagnosed MSM. A, Neighbor-joining phylogenetic tree for 131 *pol* sequences and relative reference sequences. B, CRF01_AE clade from the phylogenetic tree shown in A. Blue triangle: Kunming native MSM; green triangle: MSM form other cities in Yunnan Province; red triangle: MSM from other provinces. The sequences of MSM or other high-risk groups previously identified in China and Yunnan were included. The scale bar indicates 5% nucleotide sequence divergence. Values on the branches represent the percentage of 1000 bootstrap replicates and bootstrap values over 70% are shown in the tree.

**Figure 3:**
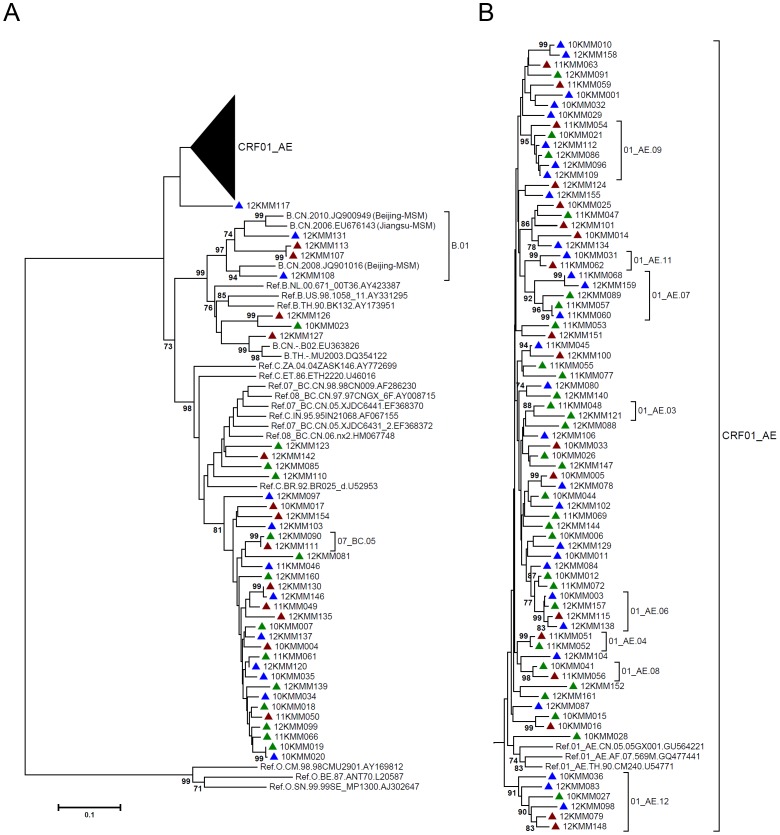
Neighbor-joining phylogenetic tree of partial *env* genes from newly HIV-1 diagnosed MSM. A, Neighbor-joining phylogenetic tree for 115 *pol* sequences and relative reference sequences. B, CRF01_AE clade from the phylogenetic tree shown in A. Blue triangle: Kunming native MSM; green triangle: MSM form other cities in Yunnan Province; red triangle: MSM from other provinces. The sequences of MSM or other high-risk groups previously identified in China and Yunnan were included. The scale bar indicates 10% nucleotide sequence divergence. Values on the branches represent the percentage of 1000 bootstrap replicates and bootstrap values over 70% are shown in the tree.

**Figure 4:**
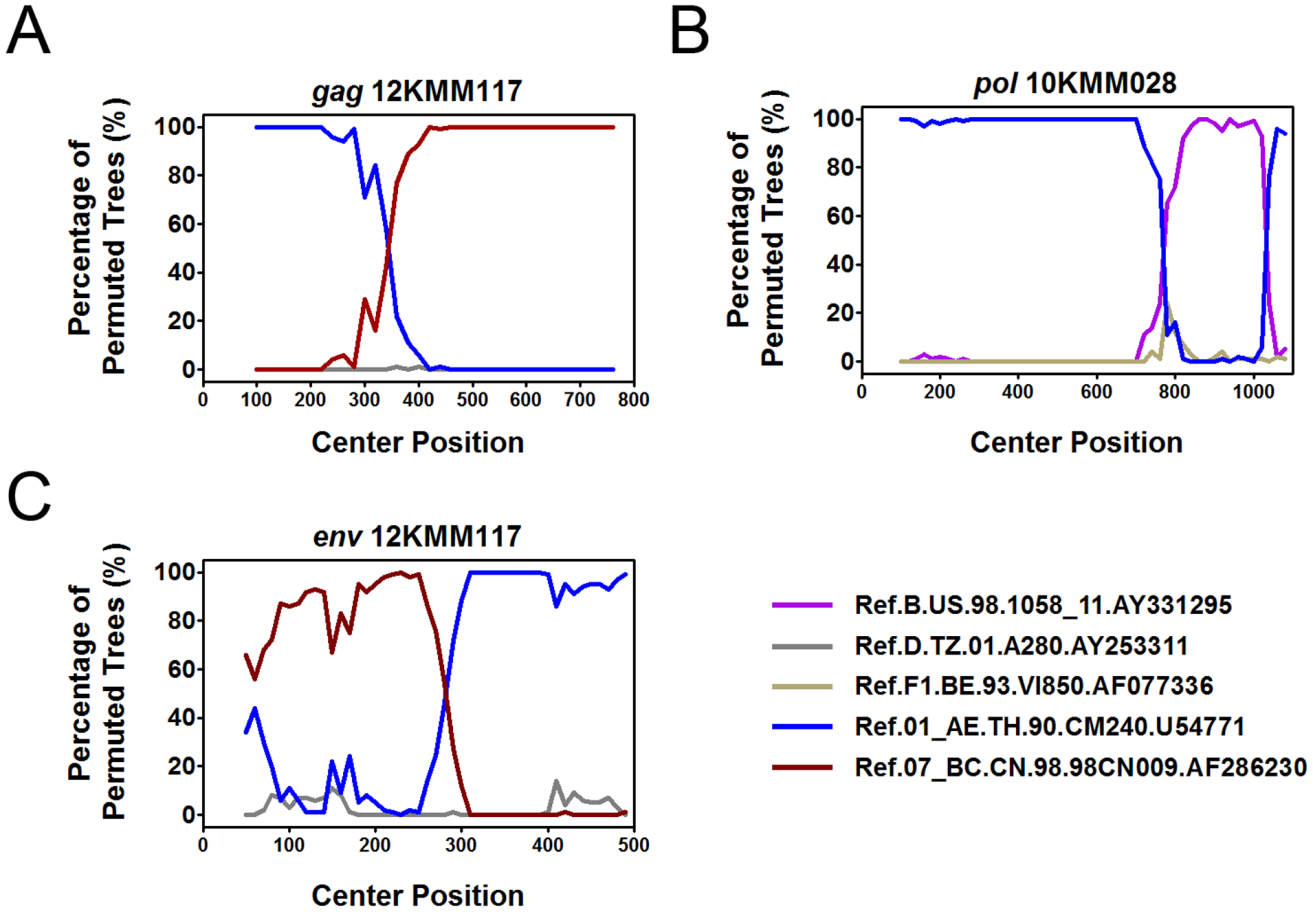
Bootscanning analysis of possible intertype mosaicism. A, Bootscanning analysis of *gag* sequences of 12KMM117. B, Bootscanning analysis of *pol* sequences of 10KMM028. The conditions used for A and B: Window: 200 bp, step: 20 bp, GapStrip: on, reps: 100, Kinura (2-parameter), T/t: 2.0. C, Bootscanning analysis of *env* sequences of 12KMM117. The condition used for C: Window: 100 bp, Step: 10 bp, GapStrip: on, Reps: 100, Kinura (2-parameter), T/t: 2.0. The reference sequences are shown at the bottom right of the figure.

For each sample, partial *gag* gene (912 bp, encoding portions of p17 and p24), *pol* gene (1197 bp, encoding protease and the first 299 residues of reverse transcriptase), and *env* gene (525 bp, encoding V3∼V4 region) were amplified and sequenced. In total, 126 *gag* sequences, 131 *pol* sequences and 115 *env* sequences were obtained, which were used to construct neighbor-joining (NJ) trees for genotyping (Figure 1–3). The potential intersubtype recombinations were confirmed by bootscanning analyses using Simplot software (Figure 4). By combining the phylogenetic tree analyses of *gag*, *pol* and *env*, a total of 131 samples generated interpretable sequence data, revealing three CRFs, one subtype, and five discrete URFs. Among the study subjects, CRF01_AE was the most common genotype (64.9%, 85/131), followed by CRF07_BC (25.2%, 33/131), URFs (5.3%, 7/131), subtype B (3.1%, 4/131), and CRF08_BC (1.5%, 2/131) (Table 1). The URFs included three CRF01_AE/B, one CRF07_BC/CRF01_AE, one CRF01_AE/CRF07_BC, one CRF01_AE/C, and one CRF07_BC/B (Table 2). The distribution of genotypes by years showed no statistical differences, however, subtype B and CRF08_BC strains were recently identified in 2012 (Table 1). The further demographic study showed that the distribution of genotypes by the participants’ permanent residence, age, nationality, marriage status, education and occupation revealed no statistical differences (Table 1).

**Table 2:** Demographic and genetic characteristics of 7 individuals with URFs.

Case Number	Area	Age	Marriage Status	*gag*	*pol*	*env*	URF
10KMM023	Yunnan Province	21	Single	CRF01_AE	CRF01_AE	B	CRF01_AE/B
10KMM028	Yunnan Province	29	Single	CRF01_AE	CRF01_AE/B	CRF01_AE	CRF01_AE/B
10KMM034	Kunming City	23	Single	CRF01_AE	CRF01_AE	C	CRF01_AE/C
12KMM088	Yunnan Province	25	Single	CRF07_BC	CRF01_AE	CRF01_AE	CRF07_BC/CFR01_AE
12KMM117	Kunming City	36	Divorced/Widowers	CRF01_AE/CRF07_BC	CRF07_BC	CRF01_AE/CRF07_BC	CRF01_AE/CRF07_BC
12KMM126	Othe Provinces	42	Single	/	CRF01_AE	B	CRF01_AE/B
12KMM131	Kunming City	34	Single	/	CRF07_BC	B	CRF07_BC/B

### Genetic Relatedness of HIV-1 Strains among MSM

As shown in the NJ phylogenetic trees of *gag* and *pol* fragments, CRF01_AE strains formed multiple discrete phylogenetic clusters with high bootstrap values (>80%) (Figure 1 and Figure 2). Most clusters included the same subjects in both *gag* and *pol* trees (Cluster 01_AE.01 to 01_AE.13). Because the reference sequences were limited, some clusters were only found in *gag* trees (01_AE.g1 and 01_AE.g2) (Figure1) or *pol* trees (01_AE.p1 and 01_AE.p2) (Figure 2). From these clusters, the genetic relatedness could be inferred. Some clusters contained sequences from MSM of other provinces, including Beijing (01_AE.10, 01_AE.11 and 01_AE.12), Liaoning (01_AE.08, 01_AE.12, 01_AE.13), Jiangsu (01_AE.11), Sichuan (01_AE.11), Zhejiang (01_AE.11), Hebei (01_AE.p1), Gansu (01_AE.p2) and Qinghai (01_AE.p2) (Figure 1 and Figure 2), suggesting a potential connection between MSM in Yunnan and the provinces described above. On the other hand, some clusters contained sequences from local heterosexually infected population (01_AE.02, 01_AE.03, 01_AE.g1 and 01_AE.g2) (Figure 1 and Figure 2), which implied a potential origin of HIV-1 strains. All of these showed multiple introductions of CRF01_AE strains into Yunnan’s MSM.

The similar situation was observed for subtype B strains in NJ trees. Three subtype B strains in Yunnan’s MSM clustered with subtype B strains from MSM in Beijing and Shijiazhuang (B.01) (Figure 1 and Figure 2). However, the other one subtype B strain (12KMM127) clustered with sequences of former plasma donors (FPDs) in Hubei Province (B.02), and also with Thailand variant of subtype B (Thai-B) (Figure 1 and Figure 3). CRF08_BC was also clustered with sequences of heterosexually infected individuals in Yunnan (08_BC.01) (Figure 1 and Figure 2), which suggested a possible derivation of CRF08_BC in Yunnan’s MSM. In the clade of CRF07_BC in *pol* tree, two clusters contained the CRF07_BC strains from MSM of Guizhou (07_BC.03 and 07_BC.04) (Figure 1 and Figure 2), a neighboring province of Yunnan, indicating the existence of a regional transmission network.

### HIV-1 Genetic Characteristics in MSM Population

To estimate the diversity of sequences belonging to different genotypes, pair genetic distances within each genotype were calculated with *gag*, *pol* and *env* sequences using the Kimura two-parameter model (Table 3). For all three gene regions, the mean genetic distances within CRF01_AE and subtype B groups were significantly larger than the counterparts within CRF07_BC group (*p*<0.05). These indicated that CRF01_AE and subtype B had more genetic diversity than CRF07_BC, which suggested two possibilities: one was multiple introductions of CRF01_AE and subtype B into the studied MSM population instead of a founder effect, the other was a longer circulating times of CRF01_AE and subtype B than that of CRF07_BC.

**Table 3:** Genetic Distances among Sequences belonging to Different Genotypes.

Gene	Genetics Distances (mean ±SE)		
	Subtype B (cases)	CRF01_AE (cases)	CRF07_BC (cases)
*gag*	0.0642±0.0149*(n = 4)	0.0430±0.0002*(n = 86)	0.0266±0.0004(n = 33)
*pol*	0.0533±0.0103*(n = 4)	0.0362±0.0002*(n = 89)	0.0210±0.0006(n = 35)
*env*	0.2257±0.0150* #(n = 7)	0.1218±0.0007*(n = 76)	0.0940±0.0026(n = 28)

* p<0.05, when comparing with the counterpart of CRF07_BC.

# p<0.05, when comparing with the counterpart of CRF01_AE.

**Figure 5:**
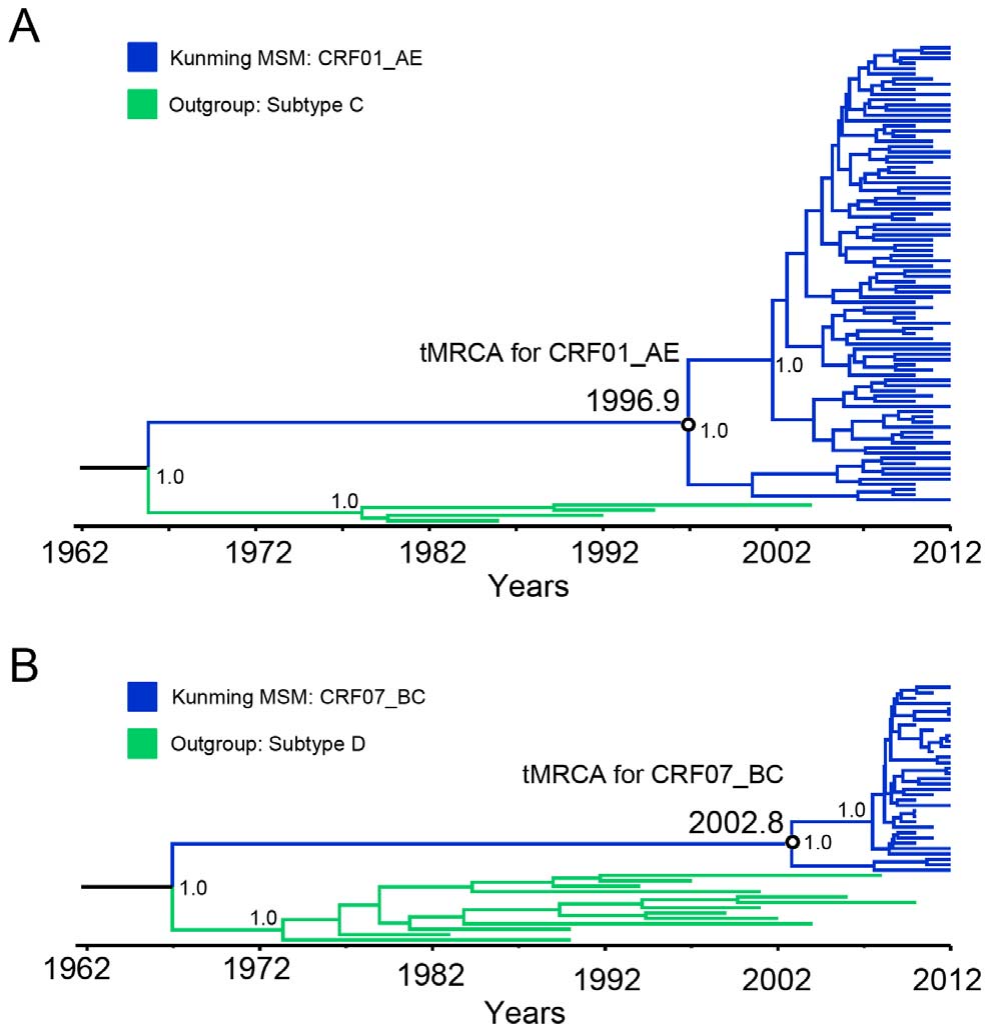
Maximum clade credibility (MCC) tree representing the rooted genealogy of CRF01_AE and CRF07_BC in Yunnan MSM. A, The MCC tree for CRF01_AE strains. HIV-1 subtype C sequences were used as outliers, including C.BR.92.U52953, C.ET.86.U46016, C.IN.95.AF067155, C.ZA.04.AY772699. B, The MCC tree for CRF07_BC strains. HIV-1 subtype D sequences were used as outliers, including D.CD.83.K03454, D.CM.01.AY371157, D.TZ.01.AY253311, D.UG.94.U88824, D.CM.10.JX140670, D.CY.06.FJ388945, D.KE.97.AY322189, D.KR.04.DQ054367, D.SN.90.AB485648, D.TD.99.AJ488926, D.UG.08.JX236672, D.YE.02.AY795907, D.ZA.90.EF633445. The MCC trees were obtained by Bayesian MCMC analysis based on partial *pol* gene (HXB2: 2147–3462) implemented in BEAST v 1.7.4. The uncorrelated exponential relaxed molecular clock method was used in combination with the Bayesian Skyline coalescent tree prior under GTR+I+G4 nucleotide substitution model. The branch lengths in the MCC trees reflect time and the corresponding time-scale is shown at the bottom of the trees. The posterior probabilities of the key nodes and the tMRCA medians for the interested nodes are indicated.

CRF01_AE and CRF07_BC were the two predominant strains circulating among MSM in Kunming. To estimate the evolutionary rates and the times of tMRCA of these two strains, MCMC analyses were performed using *pol* sequences. Based on marginal likelihood, the uncorrelated exponential relaxed molecular clock model with Bayesian Skyline coalescent tree priors was selected as the best model. The mean estimated evolutionary rates of CRF01_AE and CRF07_BC were 3.70×10^−3^ and 1.70×10^−3^ substitutions site^−1^ year^−1^ under relaxed exponential clock model. Under these substitution rates, the tMRCA for CRF01_AE and CRF07_BC circulating among MSM in Kunming were 1996.9 and 2002.8, which suggested that CRF01_AE was transmitted into MSM earlier than CRF07_BC in Kunming (Figure 5A and B).

### Genotypic Analysis of Drug Resistance

*pol* gene regions were screened for TDR-associated mutations. Among the 131 newly diagnosed and ART-naive HIV-infected MSM, six (4.6%) were identified to harbor viruses with single or multiple drug-resistant (DR) mutations (Table 4). The proportion of sequences with resistance to nucleoside reverse transcriptase inhibitors (NRTIs), non-nucleoside reverse transcriptase inhibitors (NNRTIs), and protease inhibitors (PIs) was 1.5% (2/131), 2.3% (3/131), and 2.3% (3/131), respectively. Besides four MSM with a single drug-resistant mutation, one from Henan Province (M127) had two DR mutations (Y181C and I54T), which conferred resistance to NNRTIs and PIs, and one from Zhejiang Province (M101) had four DR mutations (M184V, T215FY, K103N and M230L), which accorded resistance to NTRIs and NNRTIs (Table 3). Except one NNRTI-relative mutation (K103N) detected in two sequences, the other DR mutations just occurred once. The six drug resistant viruses showed different levels of resistance to 19 antiretroviral drugs (Table 5). Low and intermediate-level resistances to six PIs (atazanavir (ATV), indinavir (IDV), lopinavir (LPV), nelfinavir (NFV), saquinavir (SQV) and tipranavir (TPV)) were detected. Strikingly, intermediate and high-level resistances to six NRTIs (lamivudine (3TC), abacavir (ABC), zidovudine (AZT), stavudine (D4T), didanosine (DDI) and entricitabine (FTC)) and four NNRTIs (efavirenz (EFV), etravirine (ETR), nevirapine (NVP) and rilpivirine (RPV)) were detected.

**Table 4:** Demographic characteristics of 6 individuals infected with a virus containing a drug resistance mutation.

Case Number	Area	Age	Marriage Status	Genotype	Drug Resistance Mutations		
					NRTI	NNRTI	PI
11KMM045	Yunnan Province	25	Single	CRF01_AE	–	–	V82A
11KMM062	Fujian Province	18	Single	CRF01_AE	–	–	M46L
11KMM063	Jiangsu Province	34	Divorced/Widowers	CRF01_AE	T69D	–	–
12KMM101	Zhejiang Province	28	Single	CRF01_AE	M184V, T215FY	K103N, M230L	–
12KMM117	Kunming City	36	Divorced/Widowers	CRF01_AE/CRF07_BC	–	K103N	–
12KMM127	Henan Province	65	Divorced/Widowers	B	–	Y181C	I54T

PIs: protease inhibitors; NRTIs: nucleoside reverse transcriptase inhibitors; NNRTIs: non-nucleoside reverse transcriptase inhibitors;

**Table 5:** The levels of resistance to commonly used protease and reverse transcriptase inhibitors.

Case Number	PIs								NRTIs							NNRTIs			
	ATV	DRV	FPV	IDV	LPV	NFV	SQV	TPV	3TC	ABC	AZT	D4T	DDI	FTC	TDF	EFV	ETR	NVP	RPV
**11KMM045**	L	S	P	I	L	I	P	S	S	S	S	S	S	S	S	S	S	S	S
**11KMM062**	P	S	P	P	P	L	S	S	S	S	S	S	S	S	S	S	S	S	S
**11KMM063**	S	S	S	S	S	S	S	S	S	S	S	P	I	S	S	S	S	S	S
**12KMM101**	S	S	P	S	S	S	S	S	H	I	I	I	L	H	S	H	I	H	I
**12KMM117**	S	S	S	S	S	S	S	S	S	S	S	S	S	S	S	H	S	H	S
**12KMM127**	L	S	P	L	P	L	L	L	S	S	S	S	S	S	S	I	I	H	I

PIs: protease inhibitors; NRTIs: nucleoside reverse transcriptase inhibitors; NNRTIs: non-nucleoside reverse transcriptase inhibitors;

ATV: atazanavir; DRV: darunavir; FPV: fosamprenavir; IDV: indinavir; LPV: lopinavir; NFV: nelfinavir; SQV: saquinavir; TPV: tipranavir; 3TC: lamivudine; ABC: abacavir; AZT: zidovudine; D4T: stavudine; DDI: didanosine; FTC: emtricitabine; TDF: tenofovir; EFV: efavirenz; ETR: etravirine; NVP: nevirapine; RPV: rilpivirine.

S: Susceptible; P: Potential low-level resistance; L: Low-level resistance; I: Intermediate resistance; H: High-level resistance.

The relationship between demographic factors and the occurrence of drug resistance in MSM population was analyzed with logistic regression (Table 6). The multivariate analyses revealed that the occurrence of drug resistance in MSM from other provinces was significantly higher than that in Yunnan’s native MSM (aOR = 13.87), and the level of drug resistance in divorced/widowed MSM was higher than that in single MSM (aOR = 9.51).

**Table 6:** Factors associated with drug resistance in MSM population by a multivariate analysis.

Variables in Logistic Equation	Total	Cases of Drug Resistance	OR	95% CI	P value
**Area**					
Yunnan Province	97	2	1.00		
Other provinces	34	4	13.87	1.60∼120.00	0.017*
**Marriage Status**					
Single	112	3	1.00		
Married	9	1	3.53	0.21∼58.55	0.378
Divorced/Widowers	10	2	9.51	1.19∼79.09	0.034*
**Education**					
Compulsory education and below	29	3	1.00		
Compulsory education above	102	3	0.14	0.02∼1.11	0.062

* <0.05.

## Discussion

In the present work, we conducted the first systematic HIV-1 molecular epidemiological and transmitted drug resistance survey among newly diagnosed and ART-naïve MSM in Kunming City of Yunnan. By analyzing HIV-1 *gag*, *env* and *pol* genes, three CRFs, one subtype, and five URFs were identified. Among them, CRF01_AE and CRF07_BC were the two predominant strains circulating in this population. Further phylogenetic analysis showed greater HIV-1 genetic variation and clustering of infections in MSM active in Yunnan. A low level prevalence of primary HIV DR was identified. However, most DR strains were transmitted from other provinces. These clear molecular features are relevant for understanding the epidemiology of HIV among MSM in Yunnan.

MSM are vulnerable to HIV infection. To understand the HIV epidemic in MSM, many epidemiological studies had been completed, and revealed some risk factors for HIV acquisition in MSM at individual level, including unprotected receptive anal intercourse, high number of male partners and high prevalence of sexually transmitted diseases (STD) [6], [8]. However, HIV epidemic in MSM is far more complicated. Because of stigma and social discrimination, Chinese MSM would not stay in their hometown where they are easily recognized by acquaintances. Most of them concentrated into metropolises, where a close social and sexual network could be easily constructed. Thus, besides individual-level risks, network-level and community-level drivers might be crucial for remaining a high HIV transmission rates in MSM populations, because larger networks provide more opportunity for exposure to varied sexual practices and HIV-positive potential partners.

Our genetic analysis proved the genetic relatedness among HIV strains. As showed in the NJ phylogenetic trees, multiple discrete clusters were found, which suggested that the potential network existed among MSM living in Kunming. In addition, some clusters contained the sequences previously identified in MSM of other provinces, including Beijing, Liaoning, Hebei, Shandong, Jiangsu, Zhejiang, Gansu, Qinghai, Sichuan and Guizhou. These implied that Kunming’s MSM were potentially connected with MSM in these provinces. However, the transmission relationship is complex, and the bi-directional dissemination of HIV strains could occur because of MSM’s migration. In this study, 34.4% of MSM were natives from Kunming, whereas the others came from other cities in Yunnan or other provinces. The high mobility of MSM contributes to the nationwide linkage of HIV epidemic in MSM, which mostly challenges the prevention of HIV among MSM. Besides traditional behavior, barrier and biomedical interventions, community interventions have plausibility and collateral prevention benefits [26]. At a community level, promoting HIV testing, active care and antiretroviral therapy of HIV-positive MSM could decrease transmission of HIV by lowering community viral load [27], [28].

In this work, subtype B was first identified among MSM in Kunming [29], [30]. European and American subtype B (US-B) strain was considered as the earliest circulating strain in Chinese MSM, with a tMRCA estimated in 1985 [31]. In a study carried out from 1998 to 2001, this subtype was first reported among MSM in Beijing [32], where MSM have more opportunities to have close and sexual contacts with partners from the Americas and Western Europe. Hence, US-B had been the predominant strain in Beijing’s MSM [33], and disseminated among MSM in the eastern provinces of China, including Liaoning, Hebei and Anhui [31], [34], [35]. Three subtype B strains identified in our work were closely related with those in MSM of Beijing and Shijiazhuang. And two of them (12KMM107 and 113) were the students from Anhui, who might bring this subtype into Kunming by commuting between Yunnan and Anhui during holiday. It was reported that Thai-B strain was transmitted into MSM from local FBDs in central China (Henan, Anhui and Hubei) where Thai-B was prevalent [31], [36]. The only identified subject infected with Thai-B strain in our work (12KMM127) was from Henan and worked in Kunming, whose sequence clustered with those of FBDs. All of these suggested that subtype B was induced into Kunming’s MSM over provinces.

In recent years, the composition of HIV-1 genotypes in Chinese MSM has changed dynamically. The percentage of subtype B decreased, but CRF01_AE and CRF07_BC showed an increasing trend, and constituted the two most common genotypes in MSM of most cities in China [31], [37]. CRF01_AE was first identified among commercial sex workers who had returned from Thailand to Yunnan, and was predominant in heterosexually transmitted population [11], [19]. CRF07_BC originated in Yunnan by recombination between Thai-B and India C, and had been the predominant strain in intravenous drug users (IDUs) [14]. Thus, it is estimated that CRF01_AE and CRF07_BC in Chinese MSM initiated through cross high-risk behaviors. In this work, CRF01_AE and CRF07_BC were the two most common genotypes in Kunming’s MSM. However, these two genotypes showed different molecular characteristics. We found that the means of genetic distance for *gag*, *pol* and *env* within CRF01_AE were larger than those within CRF07_BC, which suggested that CRF01_AE showed more genetic diversity than CRF07_BC, and could have been separately introduced into local MSM population. Further, MCMC analysis indicated that tMRCA for CRF01_AE (1996.9) was earlier than that for CRF07_BC (2002.8). Thus, CRF07_BC is a newer strain circulating in Kunming’s MSM.

In China, the estimated prevalence of bisexual behavior among MSM is 31.2% [38], and 17.0% are married to a woman [39]. In this study, we found some CRF01_AE and CRF08_BC sequences clustered with the counterpart sequences from heterosexually infected individuals in Yunnan, which provided the evidence that bisexual behavior existed in Kunming’s MSM. Importantly, CRF08_BC was rarely reported in Chinese MSM, but was the most common genotype among recently infected heterosexually transmitted individuals in Yunnan [21]. The bisexual behavior might mediate CRF08_BC into MSM population, which could lead to another new HIV epidemic among these men.

The coexistence of multiple HIV-1 genotypes in MSM provides the prerequisite for new recombination. Compared with the previous studies, the increased frequency and types of URFs were found in our study, which made HIV-1 genetics in MSM intricate [29]–[31]. In the recent studies, URFs were also identified among MSM from other cities, some of which may be potential CRFs [31]. Thus, the characteristics of these URFs will be our research focus in the future, which will elucidate the properties of HIV-1 molecular evolution and disclose the linkage of HIV-1 epidemic among MSM.

The prevalence of primary genotypic drug resistance varied widely among Chinese MSM [22], [33], [34], [40], [41]. The high TDR rates had been reported among MSM in Beijing (15%) [33] and Shenzhen (14.6%) [40]. Our survey disclosed an estimated transmission rate of drug resistant HIV-1 strains at 4.6% among ART-naïve MSM in Kunming, which belonged to a low level of drug resistance transmission, and was equivalent to an overall TDR rate in the ordinary population in Kunming [42]. Previous studies demonstrated the majority of DR mutations among Chinese MSM were associated with PIs, which are not included in the first-line ART and of limited use in China [22], [34]. Thus, it was estimated that these PI-DR strains were derived from foreign countries with prolonged ART experience. In this study, we found that the composition of TDR associated mutations changed. The mutations to RTIs (3.8%) were higher than to PIs (2.3%). In addition, intermediate to high-level RTI resistances and low to intermediate-level PI resistances were detected. However, we did not identify a predominant DR mutation. These suggested that DR strains are not limited to internal dissemination among MSM, but there might be new DR strains entering this population. A concern is that the prevalence of RTI resistant strains could compromise the first-line antiretroviral regimens. In the multivariate logistic regression, we found that MSM from other provinces and divorced/widowed MSM were independently associated with a higher TDR rate. The former hinted that most DR strains could import from other provinces; the latter hinted that MSM with bisexual experience might be easily infected with DR strains. Kunming has been selected as a pilot site for mass screening and treatment among MSM. Our study revealed the characteristics of TDR among Kunming’s MSM, which is necessary for the evaluation and implement of this prevention measure.

Our experiment-based study provided some clues and insights in the characteristics of HIV-1 epidemic in local MSM. However, a confirmation by case interview or epidemiological survey is required. The lack of the follow-up survey after laboratory work is one limitation of this study, and will be a focus of future study.

In conclusion, our study elucidated the complicated and distinctive HIV-1 genetics among MSM population in Kunming. The phylogenetic analysis further disclosed that HIV epidemic was not limited in local MSM, but coupled with the nationwide transmission networks because of MSM’s special behavior feature. The baseline level and characteristics of transmitted drug resistance among Yunnan’s MSM was described here for the first time. These findings provide important information to develop effective measures for HIV prevention, treatment and care in this population.

## Supporting Information

Table S1 **Reference sequences used in phylogenetic anlyses.** The Access Number/Sequence ID of 54 reference sequences from MSM, heterosexually transmitted population or FBD were listed together with the relevant references.
(DOC)
