# Supplementary material for: HIV-1 Genetic Characteristics and Transmitted Drug Resistance among Men Who Have Sex with Men in Kunming, China
Source: PLoS One. 2014 Jan 29;9(1):e87033. doi: 10.1371/journal.pone.0087033 (PMC3906090; doi:10.1371/journal.pone.0087033)
Supplement: Table S1 — Reference sequences used in phylogenetic anlyses. The Access Number/Sequence ID of 54 reference sequences from MSM, heterosexually transmitted population or FBD were listed together with the relevant references. (DOC) [file pone.0087033.s001.doc]

**Table S1. Reference sequences used in phylogenetic anlyses.**

| **Gene** | **Access Number/Sequence ID** | **Population** | **Province or Country** | **Reference** |
| --- | --- | --- | --- | --- |
| ***gag*** | JQ900849, JQ900856, JQ900870, JQ900888, JQ900889, JQ900890, JQ900910, JQ900916, JQ900920, JQ900923, JQ900932, JQ900941 | MSM | Beijing |  |
|  | JX960611, JX960615, JX960635 | MSM | Liaoning |  |
|  | HQ198030 | FBD | Hubei |  |
|  | HQ215556, HQ215568 | MSM | Shijiazhuang |  |
|  | JF932499 |  |  |  |
|  | AY945710 |  | Thailand |  |
|  | YNKM09R082, YNKM09R086, YNKM09R089, YNKM09R127, YNKM09R157, YNKM09R191, YNKM09R200, YNKM09R211, YNKM09R250 | Heterosexually transmitted population | Yunnan |  |
| ***pol*** | EU921958, EU921960, EU921963, EU921977, EU921983 | MSM | Beijing |  |
|  | AY180905 |  | Henan |  |
|  | DQ990880 | FBD | Hubei |  |
|  | U71182 |  | Yunnan |  |
|  | DQ354118 |  | Thailand |  |
|  | FJ469732 |  |  |  |
|  | BJ08015, BJ08036 | MSM | Beijing |  |
|  | GS10008, GS10012 | MSM | Gansu |  |
|  | HB10018 | MSM | Hebei |  |
|  | JS09026 | MSM | Jiangsu |  |
|  | LN09012, LN09038 | MSM | Liaoning |  |
|  | QH09001 | MSM | Qinghai |  |
|  | SC08042 | MSM | Sichuang |  |
|  | SD09008 | MSM | Shandong |  |
|  | ZJ09003 | MSM | Zhejiang |  |
|  | GZ10001, GZ10031, GZ10044 | MSM | Guizhou |  |
| ***env*** | DQ354122 |  | Thailand |  |
|  | EU363826 |  |  |  |
|  | EU676143 | MSM | Jiangsu |  |
|  | JQ900949, JQ901016 | MSM | Beijing |  |

MSM: men have sex with man; FBD: former blood donor.

**References**

1. Li L, Han N, Lu J, Li T, Zhong X, et al. (2012) Genetic characterization and transmitted drug resistance of HIV-1 epidemic in men who have sex with men in Beijing, China. AIDS Res Hum Retroviruses.

2. An M, Han X, Xu J, Chu Z, Jia M, et al. (2012) Reconstituting the epidemic history of HIV strain CRF01_AE among men who have sex with men (MSM) in Liaoning, northeastern China: implications for the expanding epidemic among MSM in China. J Virol 86: 12402-12406.

3. Li Y, Takebe Y, Yang J, Zhang W, Yang R (2011) High prevalence of HIV type 1 subtype B' among heterosexuals in Western Hubei, Central China: bridging the epidemic into the general population. AIDS Res Hum Retroviruses 27: 1025-1028.

4. Li L, Lu X, Li H, Chen L, Wang Z, et al. (2011) High genetic diversity of HIV-1 was found in men who have sex with men in Shijiazhuang, China. Infect Genet Evol 11: 1487-1492.

5. Li Z, He X, Wang Z, Xing H, Li F, et al. (2012) Tracing the origin and history of HIV-1 subtype B' epidemic by near full-length genome analyses. AIDS 26: 877-884.

6. Watanaveeradej V, Benenson MW, Souza MD, Sirisopana N, Nitayaphan S, et al. (2006) Molecular epidemiology of HIV Type 1 in preparation for a Phase III prime-boost vaccine trial in Thailand and a new approach to HIV Type 1 genotyping. AIDS Res Hum Retroviruses 22: 801-807.

7. Chen M, Yang L, Ma Y, Su Y, Yang C, et al. (2013) Emerging Variability in HIV-1 Genetics among Recently Infected Individuals in Yunnan, China. PLoS One 8: e60101.

8. Wang W, Jiang S, Li S, Yang K, Ma L, et al. (2008) Identification of subtype B, multiple circulating recombinant forms and unique recombinants of HIV type 1 in an MSM cohort in China. AIDS Res Hum Retroviruses 24: 1245-1254.

9. Feng FM, Bao ZY, Zhuang DM, Liu SY, Li L, et al. (2004) [Cloning and characterization of a full-length HIV-1 genome of a prevalent subtype B-Thai strain in Henan Province]. Zhonghua Shi Yan He Lin Chuang Bing Du Xue Za Zhi 18: 356-359.

10. Tan JX, Kang XJ, Zhang W, Liu PP, Tong X, et al. (2007) Full-length clone and characterization of a human immunodeficiency virus type 1 subtype B' isolated from Hubei Province, China. Chin Med J (Engl) 120: 831-833.

11. Graf M, Shao Y, Zhao Q, Seidl T, Kostler J, et al. (1998) Cloning and characterization of a virtually full-length HIV type 1 genome from a subtype B'-Thai strain representing the most prevalent B-clade isolate in China. AIDS Res Hum Retroviruses 14: 285-288.

12. Kijak GH, Tovanabutra S, Sanders-Buell E, Watanaveeradej V, de Souza MS, et al. (2007) Distinguishing molecular forms of HIV-1 in Asia with a high-throughput, fluorescent genotyping assay, MHAbce v.2. Virology 358: 178-191.

13. Wang YE, Li B, Carlson JM, Streeck H, Gladden AD, et al. (2009) Protective HLA class I alleles that restrict acute-phase CD8+ T-cell responses are associated with viral escape mutations located in highly conserved regions of human immunodeficiency virus type 1. J Virol 83: 1845-1855.

14. Yang J, Xing H, Niu J, Liao L, Ruan Y, et al. (2012) The emergence of HIV-1 primary drug resistance genotypes among treatment-naive men who have sex with men in high-prevalence areas in China. Arch Virol.

15. Chong H, Hong K, Zhang C, Nie J, Song A, et al. (2008) Genetic and neutralization properties of HIV-1 env clones from subtype B/BC/AE infections in China. J Acquir Immune Defic Syndr 47: 535-543.

16. Guo H, Wei JF, Yang H, Huan X, Tsui SK, et al. (2009) Rapidly increasing prevalence of HIV and syphilis and HIV-1 subtype characterization among men who have sex with men in Jiangsu, China. Sex Transm Dis 36: 120-125.
